# Changes in microcirculation variables in an acute endotoxaemic equine model

**DOI:** 10.1111/evj.14473

**Published:** 2025-01-22

**Authors:** Philipp K. Sauter, Barbara Steblaj, Sabine B. R. Kästner, Franz J. Söbbeler, Julia K. Reiners, Annette P. N. Kutter, Alvaro J. Gutiérrez Bautitsta, Stephan Neudeck

**Affiliations:** ^1^ Clinic for Horses, University of Veterinary Medicine Hannover, Foundation Hannover Germany; ^2^ Section of Anaesthesiology, Vetsuisse Faculty University of Zurich Zurich Switzerland; ^3^ Clinic for Small Animals, University of Veterinary Medicine Hannover, Foundation Hannover Germany; ^4^ Tierärztliche Klinik für Kleintiere am Kaiserberg Duisburg Germany

**Keywords:** endotoxaemia, equine anaesthesia, horse, microcirculation, side‐stream darkfield technique

## Abstract

**Background:**

Microcirculation is the essential link between macrocirculation and cellular metabolism.

**Objectives:**

To test our hypotheses that microcirculation variables will show a heterogeneous flow pattern during experimental endotoxaemia, and that fluid therapy and noradrenaline (NA) infusion will normalise altered microcirculation variables.

**Study design:**

In vivo experiments.

**Methods:**

Six healthy adult horses were anaesthetised with dexmedetomidine, ketamine, and diazepam and were mechanically ventilated under isoflurane anaesthesia. Endotoxaemia was induced with 30 ng kg^−1^ *Escherichia coli* lipopolysaccharide intravenously. One hundred and twenty minutes later fluid bolus and noradrenaline (NA) infusion were administered to produce normotension. Pulse rate (PR) and mean arterial blood pressure (MAP) were measured and microcirculation variables were obtained by side‐stream darkfield technique (de Backer density (DBD), perfused de Backer density (PDBD), proportion of perfused vessels, microvascular flow index (MFI), heterogeneity index (HI)), laser Doppler flowmetry (blood flow) and white light spectrometry (tissue oxygen saturation (tSO_2_)) in sublingual, jejunal and genital area. Measurements were obtained at baseline, after endotoxin, at 60 and 120 min and during the normotensive phase. Data were analysed by mixed model variance analysis and Tukey–Kramer.

**Results:**

The PPV decreased significantly over time by 30% (*p* < 0.001) at the jejunum. MFI decreased from baseline to ET60 and from baseline to ET120 in sublingual and genital mucosa (2.9 vs. 1.4, *p* < 0.001 and 2.8 vs. 1.9, *p* < 0.01), respectively. The sublingual HI increased from baseline to ET60, ET120 and NA (0.1 vs. 0.9, *p* = 0.02; vs. 0.6, *p* = 0.01; vs. 0.3, *p* = 0.01), respectively. The genital HI increased from baseline to ET120 (0.2 vs. 1.1, *p* ≤ 0.01) and NA (0.16 vs. 0.53, *p* < 0.05, respectively). Moderate agreement between observers for MFI assessment was present (kappa = 0.4). The PR significantly increased, and MAP significantly decreased from baseline over time.

**Main limitations:**

The obtained data could be influenced by secretions, pressure artefacts, the experience of the examiner and the sampling location. Blood flow was not quantified and there was no control group.

**Conclusions:**

Overall, short‐term experimental endotoxaemia did negatively alter MFI and HI; however, it did not alter tSO_2_, blood flow, DBD, PDBD or proportion of perfused vessels. Intravenous fluid therapy and NA did not restore MFI and HI to baseline values.

## 1 INTRODUCTION

The microcirculation, which includes vessels less than 100 μm in diameter, is considered the most important site for the exchange of oxygen and nutrients between blood and tissue.^1^ Bacterial endotoxins play an important role in microcirculatory dysfunction by triggering the release of pro‐inflammatory mediators.^2^ In this context, there may be endothelial dysfunction, neutrophil–endothelial cell adhesion,^3^ activation of the coagulation system with the formation of microthrombi^4^ with functional shunt and failure of autoregulatory mechanisms.^5^ As a result, microcirculatory alteration includes changes, such as decreased vascular density, and altered blood flow.^6^ In the tissues, metabolism is reduced due to decreased perfusion. This is reflected in impaired tissue oxygen consumption.

There are two determinants to characterise blood flow, namely homogeneity and velocity of blood flow. The homogeneity of blood flow plays a significant role in the oxygen supply to the tissues. In the septic state, perfusion in microvessels exhibits a more heterogeneous flow. Consequently, blood flow may be slowed down or stopped. Capillaries with interrupted blood flow can be present right next to perfused capillaries. Despite heterogenous blood flow during septic conditions, a hyperdynamic blood flow in capillaries is well described.^1^, ^7^ Additionally, the density of perfused vessels decreases.^8^ Regarding the blood flow velocity, this determinant appears to be secondary compared with blood flow homogeneity. This results from the fact, that the regulatory mechanisms of tissue cells respond to different flow velocities by adjusting the rate of oxygen extraction.^9^

There are various devices and techniques on the market to assess and visualise microcirculation. The side‐stream darkfield camera has been successfully used on horses,^10^ cats^11^ and dogs^12^, ^13^, ^14^ on the intestinal mucosa and the sublingual and/or gingival mucosa. The side‐stream darkfield technique allows real‐time observation of microcirculatory changes, especially heterogeneous flow patterns and is a sensitive and noninvasive method. With the O2C™ device relative blood flow and oxygen saturation in tissues are measured with laser Doppler flowmetry and white light spectrometry respectively, as described elsewhere.^15^ This technique has been successfully applied to experimental horses.^16^, ^17^, ^18^, ^19^

The present study aims to show, how early endotoxaemia affects microcirculation in the horse by direct visualisation of peripheral microcirculation using the side‐stream darkfield camera and a combination of white light spectrometry and laser Doppler spectroscopy. We hypothesised that microcirculation variables will show a heterogeneous flow pattern during experimental endotoxaemia, and that fluid therapy and noradrenaline (NA) infusion will normalise altered microcirculation variables.

## 2 MATERIALS AND METHODS

### 2.1 Animals

Six horses (5 warmblood horses, 1 Thoroughbred; 2 geldings and 4 mares), American Society of Anesthesiologists classification status II, were included in the study. Horses had a body weight of 568 kg ± 65 kg (mean ± SD) and were 11.5 ± 5.3 years old. The horses were owned by the equine clinic of the University of Veterinary Medicine Hanover, Foundation, and were kept at the research facility with daily access to pasture. The horses were considered healthy in terms of cardiovascular and intestinal function according to clinical, haematological, chemical and faecal examinations, but with variable untreatable orthopaedic problems. This study investigated the effects of induced endotoxaemia on microcirculation and tissue oxygenation variables, while a parallel study focused on glycocalyx shedding and transcutaneous echocardiography. A power analysis was performed for the concurrent study on syndecan‐1 values collected from dogs with induced septic shock resulting in a size of six horses with a power of 80% and alpha of 5%.^20^ After completion of both studies, the horses were euthanised under general anaesthesia without regaining consciousness using pentobarbital 60 mg kg^−1^ bodyweight IV.

### 2.2 Study design

The study was conducted as an in vivo terminal experiment.

### 2.3 Instrumentation and anaesthesia

Food and water were provided until the morning of the experiment. A bolus of 5 μg kg^−1^ dexmedetomidine (Dexdomitor® 0.5 mg/mL; Orion Pharma) was administered intravenously (IV). Hair over the left jugular vein was trimmed and the skin was aseptically prepared. After subcutaneous infiltration of lidocaine (lidocaine hydrochloride 2%, Bela‐Pharm GmbH & Co. KG), a 12‐G intravenous catheter (Intraflon 2, 12 G‐80 mm, Vygon GmbH & Co. KG) was placed into the vein and stitched to the skin. After aseptical preparation, a port system (Exacta, 8.5 Fr; Argon Medical Devices, Inc.) for a pulmonary catheter (Balloon wedge pressure catheter, 7 Fr, 160 cm or TVEC catheter 7 Fr, 200 cm, Gaeltec) was placed in the right jugular vein. The pulmonary catheter was then inserted into a pulmonary artery by observing the site‐specific pressure curves in the standing horse.

Induction of general anaesthesia was performed with 2.5 mg kg^−1^ ketamine (Narketan® 100 mg/mL, Vetoquinol GmbH) and 0.05 mg kg^−1^ diazepam (Ziapam® 5 mg/mL, Ecuphar) IV. The horses were intubated endotracheally (Cuffed Endotracheal Tube ET 26‐30 mm, Surgivet®, Smiths Medical PM, Inc.) and placed supine on a padded operating table. Intermittent positive pressure ventilation was initiated with a positive inspiratory pressure of 20–25 cmH_2_O (Vet.‐Tec. model JAVC 2000, J.D. Medical Distributing Company, USA). Isoflurane (Isoflurane CP®, cp‐pharma) in 100% oxygen was administered to maintain anaesthesia (1.3–1.5 volume % end‐tidal concentration). The respiration rate was adjusted to maintain end‐tidal partial pressure of carbon dioxide of 35–45 mmHg. A 5 mL kg^−1^ h^−1^ Ringer's lactate solution (B. Braun Melsungen AG) and 0.33 μg kg^−1^ h^−1^ dobutamine (Dobutamine Liquid Fresenius 250 mg mL^−1^, Fresenius Kabi Deutschland GmbH) were infused. An arterial catheter (VenocanTM PLUS IV Catheter 22G 33 mm, KRUUSE A/S) was placed aseptically into the facial artery. The arterial and pulmonary catheters were connected to a fluid‐filled, low compliance extension line and a pressure transducer (Argon Safedraw Transducer; Argon Medical Devices, Inc.) placed at the level of the glenohumeral joint and zeroed to atmospheric pressure.

Anaesthesia monitoring (Datex‐Ohmeda Cardiocap/5, GE‐Healthcare Clinical Systems) included 3 lead ECG (2nd lead), heart rate, arterial and pulmonary blood pressures, pulse oximetry, peripheral oxygen haemoglobin saturation, respiratory rate, capnography, inspired and expired isoflurane concentration and temperature.

### 2.4 Surgery

A standard pre‐umbilical median laparotomy was performed in dorsal recumbency. Terminal jejunum was exteriorised 1 m orally to the ileum. The examined intestinal segment was fixed with two 40 cm long Bühner bands (Scheidenband nach Bühner 5 m, WDT) carefully placed around the intestine through a small incision through the mesentery and attached to the surgical drape using a Backhaus clamp. Regularly at 5‐min intervals, the intestine was moistened with 0.9% sodium chloride solution. Between the predetermined measurement time points, the complete intestine was placed in the abdominal cavity, and the abdominal incision was closed with clamps.

### 2.5 Endotoxaemia

Endotoxaemia was induced by 30 ng kg^−1^ lipopolysaccharide (*E. coli* B55:O5 LPS) (L2880‐10MG, Merck KGaA) diluted in 1000 mL 0.9% saline and infused IV over 30 min.

### 2.6 Fluid bolus and vasopressor

Two hours after the end of endotoxin infusion a fluid bolus of lactated Ringer's solution (Ringer's lactate, B. Braun Melsungen AG) at 10 mL kg^−1^ over 20 min was given via an infusion pump (MCP IP65, Ismatec). After completing the measurements, vasoconstriction was evoked by NA IV. The target mean arterial blood pressure (MAP) was defined to be 80–90 mmHg (normotensive phase). A starting dose of 0.1 μg kg^−1^ min^−1^ NA (Arterenol® 1 mg mL^−1^, Sanofi‐Aventis GmbH) was raised by up to 0.8 μg kg^−1^ min^−1^ to achieve the predetermined MAP.

### 2.7 Measurements

See Figure 1.

**FIGURE 1:**
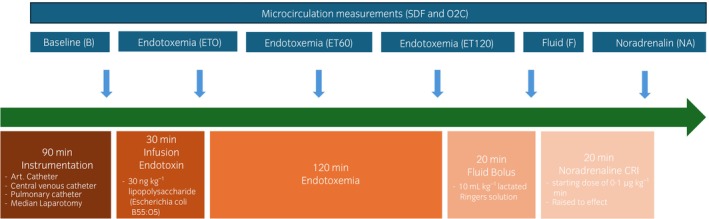
Timeline of the experiment with instrumentation, infusion of endotoxin and measurements in blue. B = baseline; ET = 0; started after ending the infusion of endotoxin; ET60 = 60 min after the end of endotoxin infusion; ET120 = 120 min after ending the endotoxin infusion; F = after 20 min of a bolus 10 mL kg^−1^ of lactated Ringer's solution; NA = after 15 min of noradrenalin infusion to maintain normotension.

### 2.8 Localisation

**FIGURE 2:**
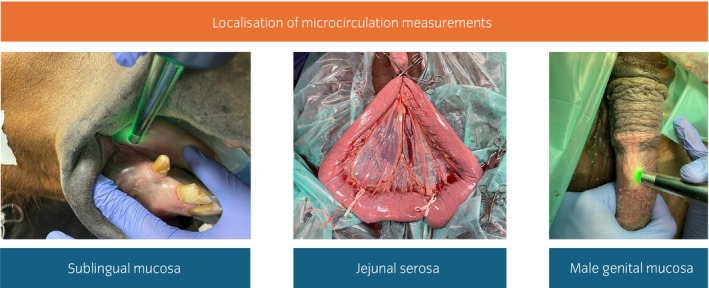
Localisation for microcirculation measurements with the side‐stream darkfield microscopy (MicroScan, MicroVision Medical) and laser Doppler flowmetry combined with white light spectrometry in six horses under general anaesthesia.

Sublingual microcirculatory measurements were performed at the tongue body. Intestinal microcirculation was determined on the antimesenteric, serosal site of the jejunum about 1 m orally to the transition into the ileum. Genital microcirculation was measured in the male horse, at an unpigmented area of the internal lamina of the preputial plica and in the mare on the inner side of the vulva 1 cm before transitioning to the outer pigmented skin layer (Figure 2).

### 2.9 Macrocirculation measurement

The pulse rate (PR) was obtained from the pulse oximeter (LifeVet® PT Pulsoximeter, Eickemeyer) placed at the tongue and MAP was recorded from the multiparameter anaesthesia monitor. Proper placement of the pulse oxymeter was confirmed by plethysmogram analysis.

### 2.10 Microcirculation measurement

#### 2.10.1 Side‐stream darkfield microscopy

Visualisation of microcirculation by the SDF camera (MicroScan, MicroVision Medical) was performed as previously described.^14^ In short, the camera was connected to a computer with analysis software. After gentle moist gauze tissue cleaning, the tip of the camera was placed on the tissue without pressure. After achieving satisfactory focus, brightness and stability, a video of 7 s was recorded. A minimum of three, but usually five, acceptable videos were analysed. Videos of insufficient quality were discarded.^1^

The de Backer density (DBD), perfused de Backer density (PDBD), and proportion of perfused vessels were analysed automatically for all vessels and vessels <20 μm. Blood vessels covered arterioles, capillaries, and venules are less than 20 μm in diameter.

The DBD defines the capillary spacing (diffusion capacity) and red blood cell velocity (convective capacity). In contrast, the PPV characterises the percentage of perfused vessels in all vessels (perfused and nonperfused).

The videos were analysed randomly by three independent and blinded examiners (PS, SN, JR) for qualitative microcirculation parameters. Microvascular flow index (MFI) is used to assess the average erythrocyte flow velocity per quadrant semi‐quantitatively as described by Pozo et al.^21^ For each quadrant, a number between 0 and 3 is assigned depending on the prevailing blood flow (0 = no flow, 1 = intermittent flow, 2 = sluggish flow, 3 = normal) and MFI represents the mean value of blood flow from all four quadrants. The ratings of the three examiners were then averaged for each time point and location. The flow heterogeneity index (HI) represents the heterogeneity of vessel flow and is calculated as (MFI maximum − MFI minimum)/mean MFI.^9^, ^22^

#### 2.10.2 Laser Doppler flowmetry combined with white light spectrometry

Laser Doppler flowmetry combined with white light spectrometry (O2C™, LEA‐Medizintechnik; software version 31.33) was used to measure microcirculation and oxygenation. In short, a fibre‐optic probe (LF‐2 probe) with a light penetration depth of 2.5 mm was applied parallel to the tissue surface and connected to a computer displaying the measurement data directly on a monitor. Measurements (performed by PS) lasted at least 30 s over four breaths.

### 2.11 Data analysis

The data were analysed using SAS Enterprise Guide statistical software (SAS Enterprise Guide Software 7.1, Inc.) and GraphPad Prism 9 statistical software (GraphPad Software, Inc.). A significance level of *p* < 0.05 was applied. The data were visually assessed using a boxplot and the distributions of the residuals on quantile‐quantile plots. Descriptive statistics used median, minimum and maximum, mean, and standard deviation. Data were analysed using a mixed model with variance analysis. The model assumption was based on a linear repeated measures model with the measurement times as influencing variables. Furthermore, a heterogeneous autoregressive model performed the correlation between time points. Subsequently, when a statistically significant difference between the measurement time points was present, pairwise comparisons were calculated using the post hoc test of Tukey–Kramer adjustment. Reliability of agreement for the three observers for the MFI values for each quadrant was calculated with Fleiss Kappa.^23^, ^24^

## 3 RESULTS

### 3.1 Macrocirculation variables

**FIGURE 3:**
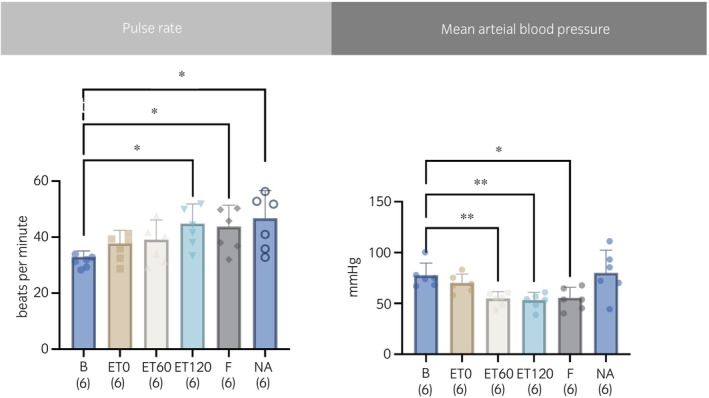
Macrocirculatory variables, pulse rate and mean arterial blood pressure obtained from six horses before (B) and 0, 60 and 120 min after intravenously induced endotoxaemia (ET0, ET60, ET120, respectively), fluid bolus (F) and noradrenaline infusion (NA) under general anaesthesia with isoflurane. Both are presented as mean (bar) and standard deviation (whiskers). The numbers in brackets represent the number of subjects with successful measurements. The dots represent each measurement from each individual. Data were analysed using mixed model with variance analysis and adjusted by Tukey–Kramer. A statistically significant result to baseline measurement is marked with an asterisk (*p ≤ 0.05; **p ≤ 0.01).

The PR was significantly increased compared with B at ET120 by 36%, at F by 33% and at NA by 42%, whereas MAP decreased significantly compared with B at ET60 by 21%, at ET120 by 24%, and at F by 21% (Figure 3).

### 3.2 Microcirculation variables

**FIGURE 4:**
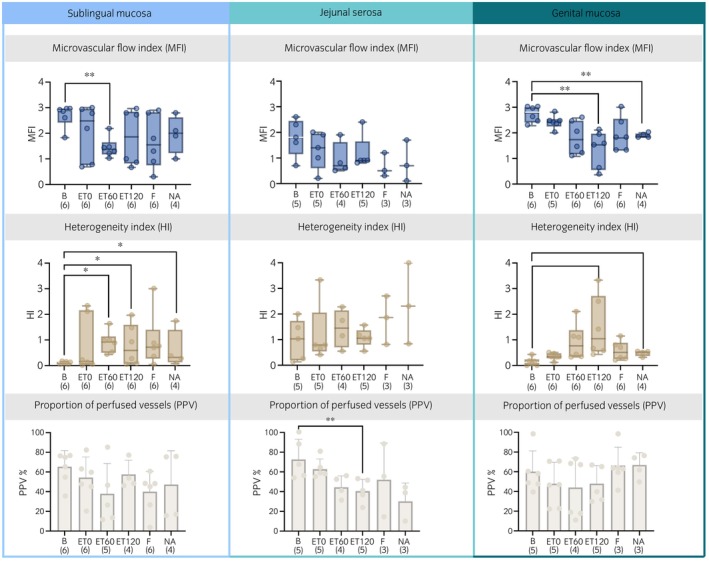
Microcirculatory variables, microvascular flow index (MFI), heterogeneity index (HI) and proportion of perfused vessels (PPV) obtained with the use of side‐stream darkfield microscopy from six horses before (B) and 0, 60 and 120 min after intravenously induced endotoxaemia (ET0, ET60, ET120, respectively), fluid bolus (F) and noradrenaline infusion (NA) under general anaesthesia with isoflurane. MFI and HI are presented as boxes (median; 25 and 75 percentile) and whiskers (min and max). Proportion of perfused vessels is presented as mean (bar) and standard deviation (whiskers). The numbers in brackets represent the number of subjects with successful measurements. The dots represent each measurement from each individual. Data were analysed using mixed model with variance analysis and adjusted by Tukey–Kramer. A statistically significant result to baseline measurement is marked with an asterisk (*p ≤ 0.05; **p ≤ 0.01).

**FIGURE 5:**
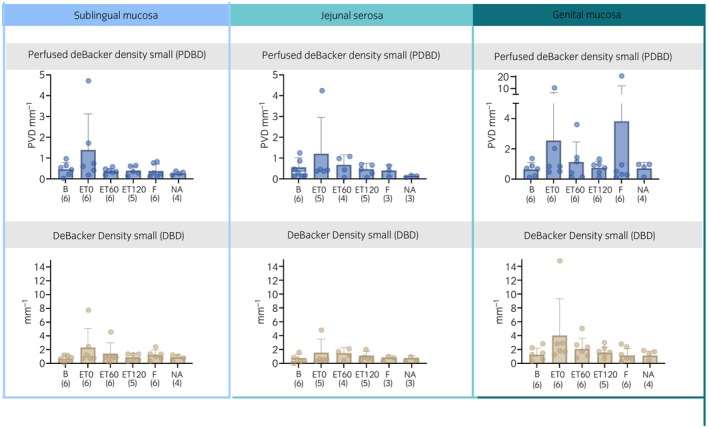
Microcirculatory variables De Backer density (DBD), perfused De Backer density (PDBD), obtained by side‐stream darkfield microscopy from six horses before (B) and 0, 60 and 120 min after intravenously induced endotoxaemia (ET0, ET60, ET120), fluid bolus (F) and noradrenaline infusion (NA) under general anaesthesia with isoflurane. De Backer Density and PDBD are presented as mean (bar) and standard deviation (whiskers). The numbers in brackets represent the number of subjects with successful measurements. The dots represent each measurement from each individual. Data were analysed using mixed model with variance analysis and adjusted by Tukey–Kramer.

Results of the microcirculation variables obtained by the side‐stream darkfield technique are presented in Figures 4 and 5. No changes over time in DBD and PDBD were detected at any location. However, proportion of perfused vessels in jejunum decreased significantly over time by 30% at time point ET120 (*p* < 0.001). A significant decrease was found for the MFI between the baseline and ET120 and time point NA on genital area (2.8 vs. 1.5, *p* < 0.01 and 2.8 vs. 1.9, *p* < 0.001, respectively). There was a significant difference between baseline MFI and ET60 (2.9 vs. 1.4, *p* < 0.001) for the sublingual area. A significant difference was found for the sublingual HI between the baseline and time point ET60 (0.1 vs. 0.9, *p* < 0.05), time point ET120 (0.1 vs. 0.6, *p* ≤ 0.01) and time point NA (0.1 vs. 0.3, *p* < 005). For the genital area a significant difference was found between baseline and time point ET120 (0.2 vs. 1.1, p ≤ 0.01) and time point NA (0.2 vs. 0.5, *p* < 0.05, respectively).

There was no significant interaction between the locations of measurements and temporal variation of variables. There was an overall significant difference between DBD at the jejunal and genital site (*p* < 0.01), and for PPV at the jejunal and sublingual site (*p* < 0.05), but no differences between any specific time point. Jejunal MFI values were significantly lower compared with sublingual (*p* < 0.01) and genital (*p* < 0.01). Both, the overall sublingual (*p* < 0.05) and genital (*p* < 0.05) HI were significantly lower as the jejunal HI.

Proper video acquisition was more difficult from the jejunal serosa due to gut motility and the moist serosa leading to movement and bubble artefacts and therefore discharge of more acquired videos. In total 10 of 36 videos were not analysed due to poor image quality for the jejunal serosa from three horses. Two videos out of 36 were not analysed due to poor image quality for the sublingual area as well as genital area of two horses each.

### 3.3 Inter‐rater reliability

Table 1 shows the Fleiss kappa with *z*‐ and *p*‐values used to evaluate inter‐rater reliability between three observers (SN, PS, JR). Four quadrants of each of the 1088 videos were assessed in a randomised fashion. A moderate agreement could be found in the total Kappa *k* = 0.4 value and values of the middle range of the scale (1 (*k* = 0.3), 2 (*k* = 0.2)). In the upper (3 (*k* = 0.6)) and lower (0 (*k* = 0.5)) fields of the scale, a considerable agreement (“substantial”) could be found.

**TABLE 1:** Fleiss Kappa to assess inter‐rater reliability between three observers (SN, PS, JR) of 1088 videos.

Numerical value	Fleiss kappa	*z*	*p*‐value
0–3 (all)	0.4	38.6	<0.001
0 (no flow)	0.5	29.80	<0.001
1 (intermittent flow)	0.3	15.71	<0.001
2 (sluggish flow)	0.2	12.33	<0.001
3 (normal flow)	0.6	32.68	<0.001

*Note*: Listed is the *z*‐value as a statistical test for significance and the *p*‐value. Listed are the total kappa value (0–3 (all)) and the individual kappa values of each numerical value with associated microvascular flow assessment in parentheses (0), (1), (2) and (3) inter‐rater reliability.

**FIGURE 6:**
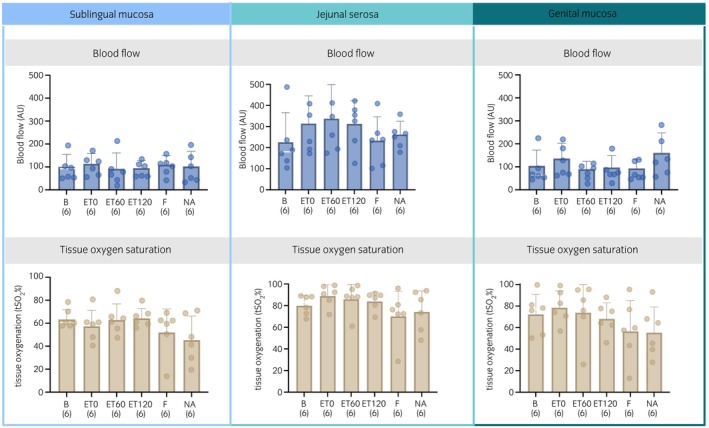
Microcirculatory variables, tissue oxygen saturation (tSO_2_) and blood flow (blood flow), obtained with the use of white light spectrometry and laser Doppler flowmetry, from six horses before (B) and 0, 60 and 120 min after intravenously induced endotoxaemia (ET0, ET60, ET120), fluid bolus (F) and noradrenaline infusion (NA) under general anaesthesia with isoflurane. Blood flow and tissue oxygen saturation are presented as mean (bar) and standard deviation (whiskers). The numbers in brackets represent the number of subjects with successful measurements. The dots represent each measurement from each individual. Data were analysed using mixed model with variance analysis and adjusted by Tukey–Kramer. AU, arbitrary unit.

Analysis of microcirculation variables obtained with white light spectrometry and laser Doppler flowmetry (Figure 6) revealed no significant difference for tissue oxygenation (tSO_2_) and blood flow in all measured areas over time. Overall, there was a significant difference between jejunal and sublingual tissue oxygenation (*p* = 0.0014). At time point ET120 sublingual tissue oxygenation was significantly lower than jejunal (64 ± 9 vs. 84 ± 9%, *p* < 0.001). Overall, blood flow was significantly higher at the jejunum compared with the sublingual (*p* < 0.001) and genital (*p* < 0.001) areas.

## 4 DISCUSSION

We hypothesised that experimental endotoxaemia will cause a heterogenous microvascular flow pattern, which will be normalised after fluids and NA infusion. Only minor changes in proportion of perfused vessels, DBD and PDBD were observed after low‐dose infusion of endotoxin in the current study. However, relevant changes in MFI and HI were observed, that did not normalise over time or after regaining normotension. The blood flow and tSO_2_ were not altered during the experiment. Global perfusion variables (PR and MAP) revealed typical early acute hyperdynamic response to endotoxaemia with tachycardia and hypotension.

Jejunal proportion of perfused vessels decreased by 30% and HI increased at the sublingual and genital area with endotoxin infusion, indicating an increase in heterogeneous flow. A decrease in proportion of perfused vessels has been observed in humans who died of sepsis or septic shock compared with survivors.^21^, ^22^, ^25^, ^26^ The reason for decreased proportion of perfused vessels is blood pooling in the capillary bed. The baseline proportion of perfused vessels values in our study appears lower in the sublingual (63%) and genital area (73%) than observed values in humans (sublingual, ~93%–99%),^7^, ^27^ cats (sublingual, ~88%),^11^ and dogs (sublingual, ~83%).^13^ These differences might be species and/or anaesthesia (protocol) related. Studies in anaesthetised horses show similar^28^ or higher baseline values (~93%).^29^ While Kieffer et al.^28^ investigated proportion of perfused vessels in horses with intestinal disease, Hurcombe et al.^29^ evaluated oral microcirculation and concluded that the site of sampling had a profound effect on the repeatability of the proportion of perfused vessels measurements. These might explain the discrepancy between the results of our and the above‐mentioned studies, as well as the difference between the three locations. In both studies, a less specific alpha‐2 agonist, namely xylazine, was used for premedication along with diazepam and ketamine for induction leading to both similar and different baseline proportion of perfused vessels values. Even though the effect of dexmedetomidine on oral and rectal microperfusion measured with the O2C™ is well described after bolus application, an effect after 90 min of bolus application seems to be unlikely due to the short elimination half‐life of 8.03 min.^19^, ^30^ The decrease in proportion of perfused vessels is supported by the observed increases in HI. Generally, a high HI is regarded as an indicator for heterogenous blood flow. Thus, it can serve as a determinant of the shunted blood fraction and indicates the presence of distributive shock.^9^ In human septic patients, HI with 0.68 was higher than in healthy controls with 0.11. There was also a difference in septic survivors and nonsurvivors with HI values of 0.45 and 1.01, respectively.^22^ We observed similar values at all measuring sites at different time points. The highest median values of HI were observed in the jejunum. However, already the baseline values were higher in the jejunal area than in the sublingual and genital area. Higher HI values in jejunum might be explained by the fact that the intestines might be more susceptible to the effect of general anaesthesia and drugs used. Opposed to the increase of HI at the sublingual and genital area after ET, the HI values in the jejunum did not increase after ET. Sublingual and genital HI continuously increased from baseline to endotoxaemia (sub. ~900%, gen. ~450%) indicating a switch from homogeneous to heterogeneous flow pattern. A reference value for HI in horses does not yet exist. Studies in humans at the skin (0.2)^31^ and sublingually (0.14)^32^ are similar to the baseline values in our horses in the sublingual (0.1) and genital (0.2) area. The assessment of the proportion of a heterogeneous flow pattern (i.e., HI) is essential for the determination of the oxygen extraction capacity in tissue.^33^, ^34^, ^35^ The baseline values of MFI recorded in the genital (2.8) and sublingual (2.9) area were comparable with those recorded in anaesthetised dogs (2.5)^13^ and horses (2.7).^28^, ^29^ Similar to the HI, baseline MFI values measured at the jejunum (1.8) were lower than in the other areas. General anaesthesia and the drugs used are known to reduce intestinal motility and intestinal blood flow. In animals, isoflurane has decreased global and microcirculatory perfusion indices.^36^, ^37^ Moreover, a decrease in MFI values by about 40%–60% after induced endotoxaemia was observed at all locations. An increased heterogeneous flow pattern and decreased flow velocity can explain the decreased MFI values. An MFI lower than 2.5 is a sign for distributive shock and a HI should be calculated.^9^

A comparison between microcirculatory perfusion variables in septic human patients demonstrated no correlation between sublingual and jejunal measurements for proportion of perfused vessels, MFI, HI^26^ and MFI.^38^, ^39^ This is similar to healthy horses (proportion of perfused vessels, MFI)^28^ and in a porcine sepsis model (proportion of perfused vessels, MFI).^40^ This contradiction can be attributable to different disease state and study designs. In horses, using the O2C™, a good correlation between sublingual and jejunal blood flow was seen.^41^ In this study, jejunal blood flow was also higher than the sublingual one, as seen in our study. However, the O2C™ measuring method laser Doppler is only able to detect differences in flow velocity rather than detecting a heterogenous flow pattern, as it is seen in sepsis. The application of darkfield microscopy for the detection of microperfusion in the equine colon failed to show a significant correlation between microperfusion (TVD, proportion of perfused vessels, PVD, MFI) and macroperfusion variables (HF, MAP), though most horses were under inotropic or vasopressor therapy.^29^

The significant increase in pulse rate is in accordance with observations in awake animals^42^ and typically seen as an early clinical sign of endotoxaemia. Most presumable this increase is a reflex due to low systemic vascular resistance which is indicated in this study by the decreased MAP. The notable decrease in mean arterial pressure (MAP) observed 60 min after the endotoxin infusion ended was likely due to endotoxin‐induced vasodilation. The mechanism underlying this vasodilation likely involves the local release of nitric oxide, which plays a crucial role as a vasodilator in both physiological and pathological processes.^43^ Vasopressor therapy aims to improve tissue perfusion pressure while avoiding excessive vasoconstriction. The vasopressor NA together with fluid therapy resulted in partial homogenisation of blood flow, which was reflected by partially improved HI values at the sublingual site, however, not significant. The NA infusion, but not the fluid bolus, improved and stabilised the MAP at 80 mmHg. This is in accordance with an observation made in septic humans, where escalating doses of NA up to 0.7 μg kg^−1^ min^−1^ to achieve a MAP of 85 mmHg did not improve sublingual microcirculation.^44^ This phenomenon, where macrocirculation variables do not reflect microcirculatory disturbances is well described in humans^1^ and also in horses,^29^ as mentioned above. Clinically this might lead to misinterpretation of the macrocirculatory variables to draw any conclusion about the perfusion status. Furthermore, Dubin et al.^44^ made an interesting finding in their study after stabilising the MAP with NA. The alteration in perfused capillary density was significantly influenced by the initial state of microcirculation. Consequently, patients with impaired sublingual perfusion at the beginning experienced an enhancement in perfused capillary density, while those with intact basal microvascular perfusion saw a decline.^44^ Contrary to our results, in nonseptic healthy horses, NA has successfully improved gastrointestinal blood flow.^45^ Additionally, there are also reports of the use of NA and the improvement of sublingual microcirculation variables in septic humans.^46^ Even though, an increase of the sublingual MFI from 2.1 to 2.4, still indicates a heterogenous flow pattern.

During a hyperdynamic sepsis event, there is a redistribution of blood flow in favour of metabolically active tissues, away from tissues with low metabolic demand.^47^ Impairments of regional perfusion would allow the assumption of locally lowered oxygen partial pressures. However, this could not be confirmed in all studies.^48^, ^49^ Immediately after infusion of the endotoxin, there was an initial, if not significant, increase in blood flow (sub. ~14%, jej. ~39%, gen. ~30%) at all three localisations, which could be interpreted as an expression of the hyperdynamic phase of endotoxaemia. This is in accordance with the concurrent study, where a significant increase in cardiac output and decrease in systemic vascular resistance index as part of the initial hyperdynamic phase of endotoxaemia could be observed. Blood flow continued to drop as the study progressed without reaching significant levels. This was improved again partially by fluid infusion and fully after administration of NA.

DBD and PDBD showed no significant change at any location over time. However, significant changes were found for DBD between the locations. Both parameters are relative values without reference data.^9^, ^14^ A trend, albeit not significant, to decreased PDBD values after an initial increase after endotoxin infusion was evident at all measurement sites. This short initial increase could indicate a hyperdynamic event typically seen in the early endotoxaemic state.^50^

When assessing the inter‐rater video assessment reliability for MFI, a fair to substantial agreement between the examiners was found.^23^ In the range of the extreme values, a higher inter‐rater reliability could be achieved than in the middle range of the scale. This could indicate that all observers could identify severely impaired or normal values better than slightly modified values. A moderate agreement (kappa = 0.5) in three categories ranging from good to poor could be found in sublingual microcirculation of endotoxaemic pigs.^51^ A human study investigating the inter‐rater variability of MFI in sepsis reported a value of 0.85. These values were higher than in the current study. However, it must be considered that the measurements in pigs were performed with the orthogonal polarisation spectral Imaging (OPS) technique.^52^ A study in pigs with side‐stream darkfield on intra‐observer variability of MFI found only low agreement with correlation coefficients of 0.52.^53^ In the former studies, the data distribution was more dispersed with OPS, while the Petersen et al. study with side‐stream darkfield had several values in the upper ranges between 2 and 3. Thus, a change from 0 to 1 in MFI may not reveal the same microcirculatory effects as a change from 2 to 3 in MFI.

One limitation of the study was the precision in the recording of videos being influenced by secretions,^13^ pressure artefacts and the experience of the examiner.^12^ The videos were recorded by the same examiner (PS) under supervision of an experienced person (BS). Satisfactory videos could not always be generated, especially at the jejunal site, leading to videos with poor image quality and fewer videos for each time point. Though, the lower baseline MFI at the jejunal site is more likely to be the result of the effect of general anaesthesia itself or intestinal peristalsis artefact as there was only one video with poor image quality. Unsatisfactory video acquisition is consistent with a previous study.^11^ Additionally, the manual video analysis for MFI is another limitation of side‐stream darkfield. Although all examiners (PS, SN, JR) were trained using literature and exemplary video sequences to perform visual analysis of MFI, this process remains complex due to the relative scale values ranging from 0 (no flow) to 3 (normal). Furthermore, this scale does not include criteria for hyperdynamic flow, often seen in the initial state of sepsis.

Repetition of the exact same localisation as in the previous measurement could not be guaranteed, due to slight movement of the examination camera to achieve acceptable video quality. Additionally, image quality at pigmented skin is poorer than at nonpigmented sites.^13^ Due to this, unpigmented sites on the genitals were preferred on the geldings. Another limitation of the study were the different localisations. The sublingual and vaginal layers contain a mucosal tissue layer, the jejunal layer includes a serosal layer, whereas, in the male animal, the integument is the basis of the tissue to be investigated. The study population consists of two geldings and four mares, which may also affect the results in the genital area.

A limitation of the O2C™ method is the specification of the blood flow in arbitrary units. Therefore, no quantitative statement about the blood flow can be made. To assess trends, a baseline measurement is performed, based on which the changes can be evaluated qualitatively.^41^, ^45^ Stray light can affect the quality of the haemoglobin spectrum and generate incorrect oxygen saturation values in the tissue.^54^ In addition, wrong values of relative blood flow and velocity may be recorded due to the movement of the probe, either by the examining person or by movement artefacts due to intestinal peristalsis.^54^ Thus, it should be mentioned that the Doppler shift generated by erythrocytes moving in the vessels can be negatively affected by the contraction of the intestine. Oxygen saturation can be analysed independently of the Doppler shift and is less susceptible to motion artefacts. Overall, microcirculation should be regarded as a dynamic process. Locally, it can be heterogeneous in time and place and thus influence the measurement result.^19^, ^54^

This study is lacking a control group. Therefore, the effect of sedatives and anaesthetic gases to induce and maintain general anaesthesia as well as anaesthesia time itself may also be considered a possible cause of microcirculatory changes. Though, inspired isoflurane concentration, dosage of sedatives and time were comparable between the probands. Dobutamine was administered to increase blood pressure and cardiac output, which were reduced by general anaesthesia and endotoxaemia. However, we did not change the dose over the study and the administration was necessary to maintain MAP above (40 mmHg) during general anaesthesia.

The present study does not provide any conclusion about the outcome due to euthanasia of the horses.

## 5 CONCLUSION

Endotoxaemia dysregulated the macrocirculation indicated by PR and MAP and microcirculation in anaesthetised clinically healthy adult horses. The microcirculation could be successfully assessed qualitatively and quantitatively using side‐stream darkfield, laser Doppler flowmetry and white light spectroscopy. The decreased perfused vessel density and the increased heterogeneous flow pattern with tissue hypoperfusion indicate early signs of septic microcirculatory disorder. Administration of crystalloid fluids and NA at the dose used did not completely resolve heterogenous blood flow.

## AUTHOR CONTRIBUTIONS

**Philipp K. Sauter:** Writing – original draft; investigation; conceptualization. **Barbara Steblaj:** Investigation; writing – original draft; writing – review and editing; resources. **Sabine B. R. Kästner:** Conceptualization; investigation; writing – review and editing; resources; supervision. **Franz J. Söbbeler:** Investigation; conceptualization. **Julia K. Reiners:** Investigation. **Annette P. N. Kutter:** Investigation; writing – review and editing; resources. **Alvaro J. Gutiérrez Bautitsta:** Investigation. **Stephan Neudeck:** Conceptualization; investigation; writing – review and editing; supervision; visualization; methodology; project administration.

## FUNDING INFORMATION

This work was not supported by any funding.

## CONFLICT OF INTEREST STATEMENT

The authors declare no conflicts of interest.

## DATA INTEGRITY STATEMENT

Philipp Sauter and Stephan Neudeck had full access to all the data in the study and take responsibility for the integrity of the data and the accuracy of data analysis.

## ETHICAL ANIMAL RESEARCH

Ethical approval was obtained from the Ethics Committee of the State of Lower Saxony, Germany (approval number 33.19‐42502‐04‐20/3481).

## INFORMED CONSENT

Not applicable.

## PEER REVIEW

The peer review history for this article is available at https://www.webofscience.com/api/gateway/wos/peer-review/10.1111/evj.14473.

## Data Availability

The data that support the findings of this study are openly available in Mendeley at http://doi.org/10.17632/ksxv9hxdwb.1.
